# Microcystin-LR Induced Apoptosis in Rat Sertoli Cells via the Mitochondrial Caspase-Dependent Pathway: Role of Reactive Oxygen Species

**DOI:** 10.3389/fphys.2016.00397

**Published:** 2016-09-09

**Authors:** Hui Huang, Chuanrui Liu, Xiaoli Fu, Shenshen Zhang, Yongjuan Xin, Yang Li, Lijian Xue, Xuemin Cheng, Huizhen Zhang

**Affiliations:** Department of Environmental Health, School of Public Health, Zhengzhou UniversityZhengzhou, China

**Keywords:** microcystin-LR (MC-LR), reactive oxygen species (ROS), mitochondria, apoptosis, Sertoli cells

## Abstract

Microcystins (MCs), the secondary metabolites of blue-green algae, are ubiquitous and major cyanotoxin contaminants. Besides the hepatopancreas/liver, the reproductive system is regarded as the most important target organ for MCs. Although reactive oxygen species (ROS) have been implicated in MCs-induced reproductive toxicity, the role of MCs in this pathway remains unclear. In the present study, Sertoli cells were employed to investigate apoptotic death involved in male reproductive toxicity of microcystin-LR (MC-LR). After exposure to various concentrations of MC-LR for 24 h, the growth of Sertoli cells was concentration-dependently decreased with an IC_50_ of ~32 μg/mL. Mitochondria-mediated apoptotic changes were observed in Sertoli cells exposed to 8, 16, and 32 μg/mL MC-LR including the increased expression of caspase pathway proteins, collapse of mitochondrial membrane potential (MMP), and generation of ROS. Pretreatment with a global caspase inhibitor was found to depress the activation of caspases, and eventually increased the survival rate of Sertoli cells, implying that the mitochondrial caspases pathway is involved in MC-LR-induced apoptosis. Furthermore, N-acetyl-l-cysteine attenuated the MC-LR-induced intracellular ROS generation, MMP collapse and cytochrome c release, resulting in the inhibition of apoptosis. Taken together, the observed results suggested that MC-LR induced apoptotic death of Sertoli cells by the activation of mitochondrial caspases cascade, while its effects on the ROS-mediated signaling pathway may contribute toward the initiation of mitochondrial dysfunction.

## Introduction

In recent years, eutrophication caused by cyanobacteria bloom events occurs frequently in the global scope. Several cyanobacterial species are known to produce a wide variety of potent toxins leading to harmful cyanotoxin contamination, which is harmful to the safety of community drinking water (Jochimsen et al., 1998; Li et al., 2011; Merel et al., 2013; Boopathi and Ki, 2014). Among all the cyanotoxins, microcystins (MCs) are the most extensively studied owing to their ubiquity and high toxicity (Campos and Vasconcelos, 2010; Puddick et al., 2014).

MCs are cyclic seven peptide compounds with stable chemical properties, and thus it is difficult to remove from water bodies by conventional water treatment processes (de la Cruz et al., 2011). Furthermore, MCs can be accumulated in aquatic organisms and be transmitted to higher trophic levels by food chain, thereby threatening public health (Meneely and Elliott, 2013). At present, more than 100 types of MCs congeners have been identified in freshwater systems, amongst which microcystin-LR (MC-LR) is the most abundant and potent variant (Gupta et al., 2003). A large number of studies have confirmed that liver is the first target organ for MC-LR with resultant hepatotoxicity (Sun et al., 2011; Li and Han, 2012; Chen et al., 2013; Wang et al., 2014; Li et al., 2015). In addition, MC-LR could induce the process of apoptosis in many cells including rat Sertoli cells, spermatogonia, and Chinese hamster ovary (CHO) cells (Zhang et al., 2011; Li and Han, 2012; Zhou et al., 2012; Chen et al., 2013; Xue et al., 2015). Gonads are the second most important target organs for MCs (Chen and Xie, 2005), and in fact, recent studies have indicated that MCs can be accumulated in male gonads of rodents, and exert adverse reproductive effects in a dose- and time-dependent manner (Wang L. H. et al., 2013; Wu et al., 2014). Sertoli cells can affect testis formation and spermatogenesis by providing nutrition and cellular morphology support for the germ cells (Griswold, 1998; Liu et al., 2014). As we all known, Sertoli cells were conducive to the construction of the blood–testis barrier (Kaur and Bansal, 2004). Therefore, the impaired Sertoli cells would be harmful to male reproduction.

Furthermore, apoptosis of cells involved in the process of spermatogenesis or oogenesis has been shown to induce deleterious effects on the reproductive system. In our preliminary studies, it was observed that MC-LR led to injury and apoptosis of Sertoli and CHO cells, chromatin condensation, nuclei fragmentation, induction of apoptosis genes (p53, bax), and activation of caspase-3 (Zhang et al., 2011; Yang et al., 2013). Recent studies have also demonstrated that MC-LR treatment could induce oxidative stress and increase expression of apoptotic cascade proteins in rat testicular cells (Li and Han, 2012; Wang X. T. et al., 2013).

MC-LR-induced cytotoxicity has been implicated in reactive oxygen species (ROS) generation due to the depletion of glutathione and protein-bound sulfhydryl groups (Bieczynski et al., 2013). ROS, mainly generated in the mitochondria, is a double-edged sword that can interact with living cells to regulate cellular functions ranging from cell proliferation to cell death (Darley-Usmar et al., 1995). Our recent study addressing acute low-dose exposure to MC-LR demonstrated its ability to induce oxidative damage *in vitro*; MC-LR decreased cell viability, reduced mitochondrial membrane potential (MMP), up-regulated antioxidant (superoxide dismutase, glutathione reductase, glutathione peroxidase) activity, and increased the production of ROS and lipid peroxidation (Xue et al., 2015). Although these results suggested that ROS might constitute a direct cause of mitochondrial dysfunction, it remains controversial as to how ROS actually functions during MC-LR-mediated Sertoli cells apoptosis.

In the present study, we investigated whether MC-LR induced apoptosis in rat primary-cultured Sertoli cells through mitochondria-dependent pathways with caspase activation. Moreover, N-acetyl-l-cysteine (NAC), a well-known antioxidant agent, is effective in protecting cells from ROS-mediated intracellular oxidative injury. However, little is known about the effects of NAC on the reproductive toxicity of MC-LR. Thus, for the first time, we also evaluated the role of NAC in the regulation of MC-LR-induced ROS-mediated apoptosis in Sertoli cells.

## Materials and methods

### Animals and reagents

Male Sprague-Dawley rats (18- to 20-Day-old) were purchased from the Experimental Animal Center of Henan province (Zhengzhou, China) and kept in accordance with the Guide for the Care and Use of Laboratory Animals published by the Ministry of Health of the People's Republic of China.

MC-LR with purity of ≥95% was purchased from Beijing Express Technology Co. (Beijing, China). NAC was purchased from Sigma-Aldrich (St. Louis, MO, USA). Cell Counting Kit-8 (CCK-8), ROS Assay Kit, MMP Assay Kit, Cell Lysis Buffer for Western blotting and immunoprecipitation (IP), Cell Mitochondria Isolation Kit, BCA Protein Assay Kit, and zVADfmk were purchased from Beyotime Institute of biotechnology (Haimen, Jiangsu, China). Caspase-3 (YT0656), cleaved-caspase-3 (ab2302), caspase-9 (YT0662), cleaved-caspase-9 (YC0012), cytochrome c (Cyt c, E2A6028), and anti-β actin (CW0096) were purchased from ImmunoWay Biotechnology Company (Newark, DE, USA). Anti-GAPDH (ab8245) and anti-Hsp70 (ab2787) were purchased from Abcam Company (Cambridge, UK). Annexin V-FITC/propidium iodide (PI) apoptosis detection kit and Trypsin were purchased from Beijing Solarbio Science & Technology Company (Beijing, China). Ultrapure water was obtained using a Milli-Q water purification system from Millipore (Bedford, MA, USA).

### Sertoli cell preparation

Sertoli cells were isolated from Sprague-Dawley rats (*Rattus norvegicus*) at the age of 18–20 days according to previous procedures with some modifications. Testes were decapsulated, minced, and washed twice in Hanks' balanced salt solution (HBSS), then digested sequentially in 10 mL HBSS with 0.25% trypsin (Solarbio, Beijing, China) and 0.1% collagenase (Solarbio, Beijing, China) in a shaking water bath (Thermo Fisher Scientific, Massachusetts, USA) at 37°C for 30 min. The digested cell suspension was washed extensively with Dulbecco's modified Eagle's medium (DMEM) without phenol red to remove peritubular cells, followed by filtration using the BD Falconcell strainers (nylon mesh size, 70 μg/mL). Finally, Sertoli cells were cultured in DMEM media (Gibco, Carlsbad, CA, USA) supplemented with 10% fetal bovine serum (FBS; Gibco, Carlsbad, CA, USA), 2 mM L-glutamine, 10 U/mL of penicillin (Solarbio, Beijing, China) and 10 μg/mL of streptomycin (Solarbio, Beijing, China) in a humidified atmosphere for 24 h at 37°C (95% air and 5% CO_2_). Then, these cells were extensively washed twice with HBSS to remove the unattached cells, before treatment with 20 mM Tris-HCl (pH 7.4) for 5 min and serum starvation for another 24 h. The purified Sertoli cells formed a monolayer in the medium.

### Cell culture and treatments

About 2 × 10^6^ Sertoli cells were incubated in 10 cm-diameter dishes. A MC-LR stock solution was dissolved in methanol to generate 1 mg/mL of stock solution and further diluted with culture medium to the desired concentrations, prior to incubation with Sertoli cells for 24 h. Additionally, the caspase inhibitor, zVADfmk, was dissolved in DMSO (Solarbio, Beijing, China) at 20 mM as a stock solution, while NAC was dissolved in PBS at 400 mM as a stock solution. The cells were pretreated with 50 μM zVADfmk or 10 mM NAC for 1 h prior to the addition of MC-LR.

### Cell proliferation assay

Cells were incubated for 24 h, followed by treatment with MC-LR at various concentrations (0, 1, 5, 10, 20, 40, 60 μg/mL) for another 24 h. The control group was incubated with vehicle control containing 0.1% v/v methanol (0.79 mg/mL; 0.1% v/v methanol had no significant cytotoxicity; Figure S1). Then the medium was removed, followed by the addition of 20 μL of CCK-8 solution (5 mg/mL) to each well and incubation for another 4 h at 37°C. Finally, 150 μL of DMSO was added to each well, and the optical density was measured at 490 nm with a Sunrise Remote microplate reader (BioTek, Highland Park, United States). Cell proliferation inhibition rate was calculated and the IC_50_ dose of MC-LR for 24 h was determined.

### Annexin V-FITC/PI staining for cell death detection

The apoptosis of Sertoli cells was tested with an apoptosis detection kit following staining with either annexin-V-FITC alone or in combination with PI according to manufacturer's instructions. In brief, 1 × 10^6^ cells in a 60 mm culture Nunc were incubated with the designated doses of MC-LR with or without 50 μM zVADfmk or 10 mM NAC for 24 h. After incubation, the cells were washed twice with cold PBS, followed by collection and re-suspension in 500 μL of binding buffer (10 mM HEPES/NaOH pH 7.4, 140 mM NaCl, 2.5 mM CaCl_2_) at a concentration of 1 × 10^6^ cells/mL. Subsequently, 5 μL each of annexin V-FITC and PI were added to each sample, followed by incubation at room temperature for 15 min in the dark. Cells were subjected to flow cytometry with a FACS Calibur flow cytometer (BD Accuri C6, New Jersey, USA).

### Determination of intracellular ROS production

Production of intracellular ROS was detected using the florescent probe 2-7-dichlorofluorescein diacetate (DCFH-DA). In this assay, cells were exposed to MC-LR and NAC for 24 h, and then co-incubated with DCFH-DA (10 μM) for 30 min at 37°C in the dark. Cells were harvested and washed with PBS, and then ROS was detected by measuring the fluorescence intensity on a FACS Calibur flow cytometer. The fluorescence intensity of the cells was also observed under a fluorescence microscope (Nikon ECLIPSE TI, Japan).

### Measurement of MMP

The MMP of Sertoli cells was measured using the MMP Assay Kit and JC-1 (Beyotime, Shanghai, China) according to the manufacturer's instructions. JC-1 is a cationic dye and can be accumulated in the membrane of mitochondria. Under normal conditions, the mitochondrial membrane shows red fluorescence; when MMP is lost, the red fluorescence decreases, with corresponding increase in green fluorescence. The intensity ratio of red to green fluorescence represents the change in MMP. Briefly, after MC-LR treatment with or without NAC for 24 h, Sertoli cells (1 × 10^6^ cells/well) were trypsinized, washed in ice-cold PBS and then stained with JC-1 (5 μg/mL) for 20 min at 37°C, followed by flow cytometry immediately with a BD FACS Calibur flow cytometer.

### Western blotting analysis

The total proteins of Sertoli cells were extracted after MC-LR exposure with or without inhibitor for 24 h. Western blotting and IP were performed to detect caspase-3, caspase-9, and cleaved-caspase-3. For the measurement of Cyt c, the cytoplasmic, and mitochondrial proteins were isolated from Sertoli cells using Cell Mitochondria Isolation Kit. Then, the protein content was measured by the BCA Protein Assay Kit. Cell extracts were separated on SDS-PAGE and transferred onto PVDF membranes (Millipore, Bedford, MA, USA). These membranes were blocked with 2.5 or 5% bovine serum albumin (BSA) in TBS-Tween 20 (Solarbio, Beijing, China) for 1 h at 37°C, and then incubated with the primary antibody (1:1000 dilution) at 4°C overnight. Finally, the membranes were treated with the HRP-coupled secondary antibodies (1:5000 dilution) for 1 h at 4°C. The membranes were washed with T-TBS after each antibody binding reaction, and detection of each protein was performed using an ECL reagents kit (Santa Cruz, Dallas, TX, USA). GAPDH was used as the cytoplasmic housekeeping control, while Hsp70 was used as the mitochondrial housekeeping control. Furthermore, to show the successful subcellular fractionation, we detected GAPDH in the mitochondrial fraction and Hsp70 in the cytoplasmic fraction. The β -actin antibody was used as the internal housekeeping control, and the blots were scanned and quantified by specific software (Image J) (National Institutes of Health, Bethesda, MD, USA).

### Statistical analysis

Data were from three independent experiments and statistical analysis was done with SPSS 21.0 (SPSS Inc., Chicago, IL, USA). One-way analysis of variance (ANOVA) was used to analyze the difference between groups. Student-Newman-Keuls test (*SNK*) was used for multiple comparison in variances with homogeneity and Dunnett-t3 test in variances without homogeneity. A value of *P* < 0.05 was considered statistically significant.

## Results

### Toxic effects of MC-LR on sertoli cells after various treatments

**Figure 1:**
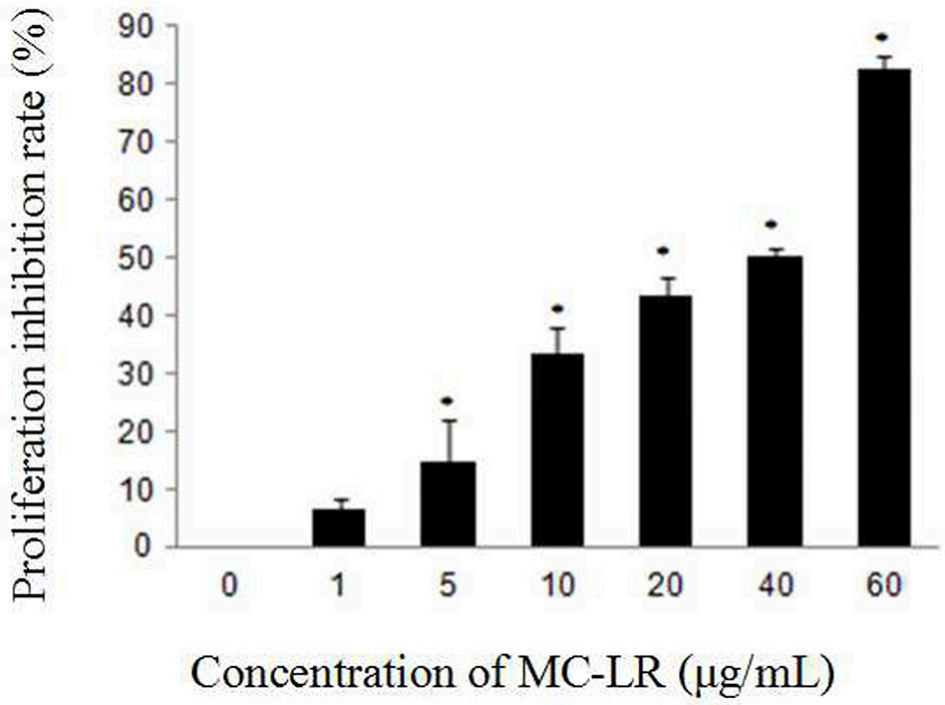
**Effect of MC-LR on the proliferation inhibition of rat Sertoli cells using CCK-8 assay**. Cells were exposed to MC-LR at different concentrations (0, 1, 5, 10, 20, 40, and 60 μg/mL) for 24 h, and the optical density (OD) was detected with CCK-8 assay. Data were presented as mean ± SEM of triplicate independent experiments. ^*^P < 0.05 vs. control group.

To evaluate the effect of MC-LR on the growth of Sertoli cells, we treated the cells with a gradient of concentration of MC-LR for 24 h. The results indicated that the cell growth was inhibited in a concentration-dependent manner (Figure 1), and the IC_50_ dose of MC-LR for 24 h was determined to be 32 μg/mL. Thus, 8, 16, and 32 μg/mL of MC-LR (IC_50_/4, IC_50_/2, and IC_50_, respectively) were used in subsequent experiments.

### MC-LR induced apoptosis in sertoli cells via the activation of caspases

**Figure 2:**
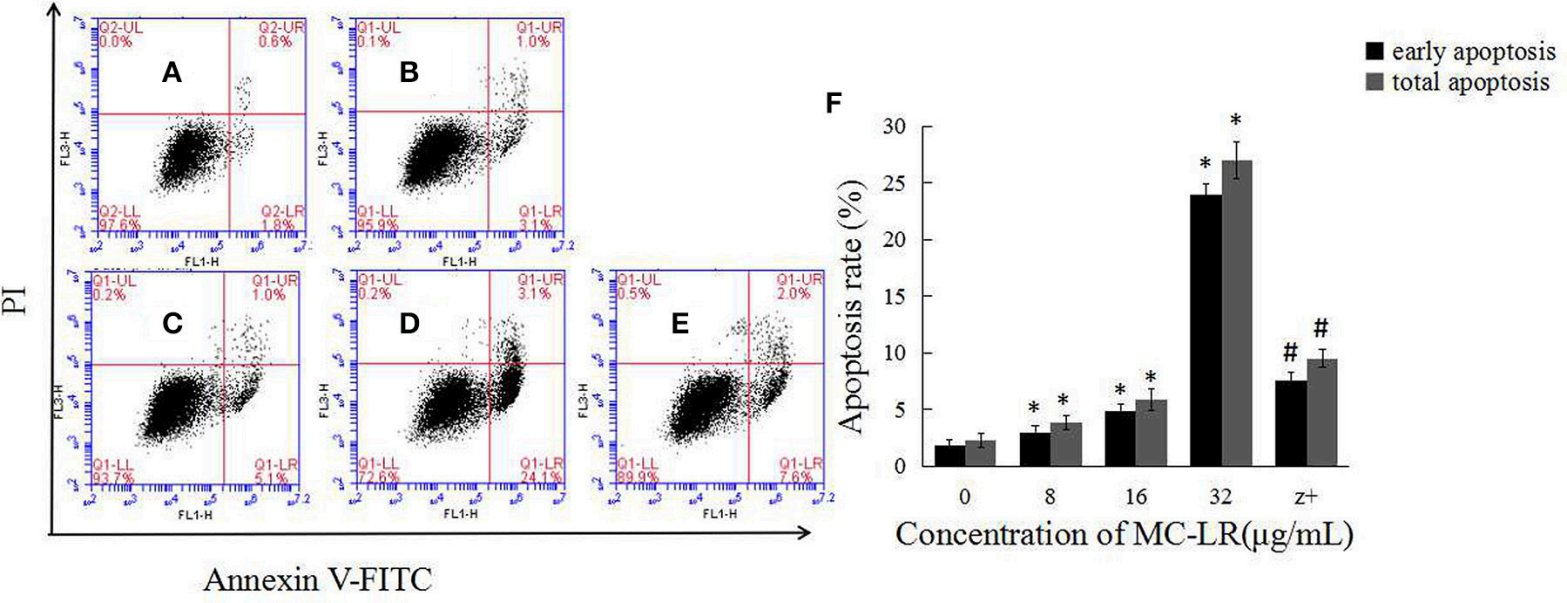
**MC-LR induced apoptosis of rat Sertoli cells**. After cells were treated with MC-LR for 24 h, cell apoptosis rate was determined by flow cytometry after annexin V-FITC/PI staining **(A–E)** and data were expressed as mean ± SEM **(F)**. **(A)** Control group; **(B)** 8 μg/mL MC-LR; **(C)** 16 μg/mL MC-LR; **(D)** 32 μg/mL MC-LR, **(E)** 50 μM zVADfmk + 32 μg/mL MC-LR (*n* = 3). ^*^P < 0.05 vs. control group; ^#^*P* < 0.05 vs. 32 μg/mL MC-LR.

To determine the apoptosis index of Sertoli cells, flow cytometry was done after annexin-V FITC and PI staining. As shown in Figure 2, the apoptosis index of Sertoli cells was significantly higher in the MC-LR group than that in the control group.

**Figure 3:**
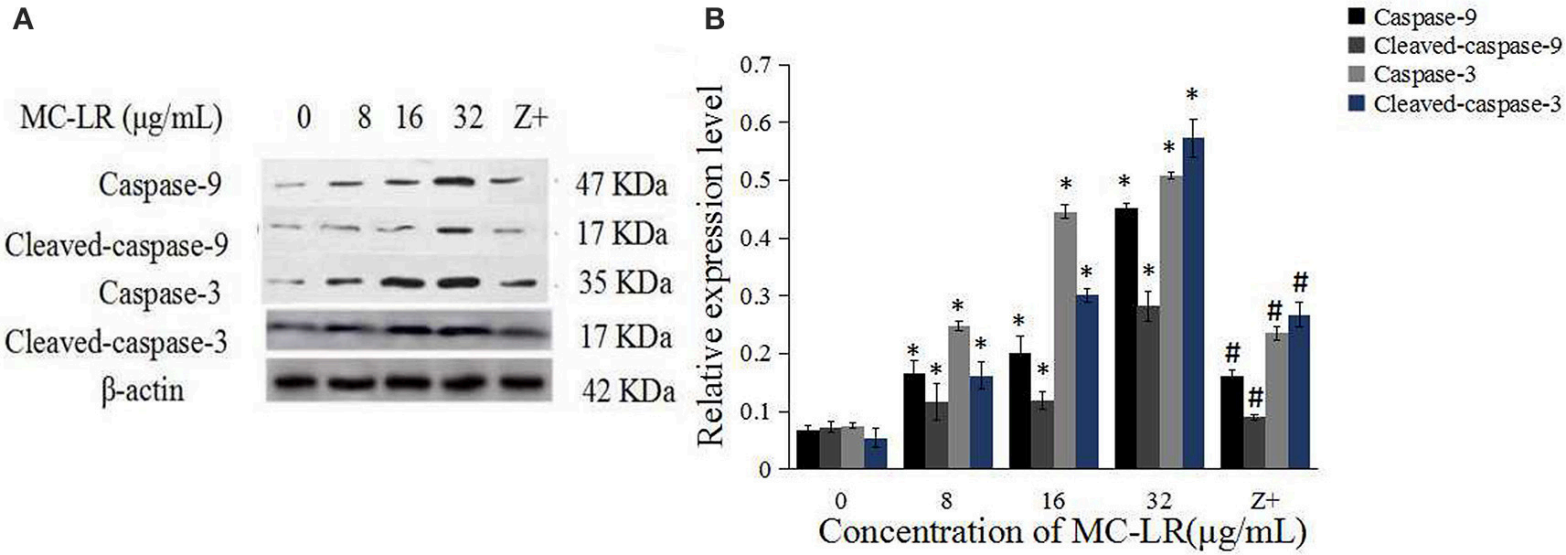
**MC-LR induced activation of caspases. (A)** Western blotting results of caspase-9, cleaved-caspase-9, caspase-3, and cleaved-caspase-3 in rat Sertoli cells after various concentrations of MC-LR and 50 μM zVADfmk + 32 μg/mL MC-LR, respectively. Z+, 50 μM zVADfmk + 32 μg/mL MC-LR. **(B)** Relative quantification analysis of **(A)**. β -actin was used as the internal control. Data were presented as mean ± SEM of triplicate independent experiments and analyzed by one-way ANOVA. ^*^P < 0.05 vs. control group; ^#^*P* < 0.05 vs. 32 μg/mL MC-LR.

The activation of caspases by various stimuli plays a crucial role in apoptotic cell death. Thus, to investigate whether MC-LR induced apoptosis of Sertoli cells through a caspase cascade-dependent pathway, cells were pretreated with 10 μM caspase inhibitor zVADfmk for 1 h and then exposed to MC-LR. Compared to the non-zVADfmk group, the apoptosis index of the groups pre-treated with zVADfmk was reduced significantly (*P* < 0.05; Figure 2E). Moreover, we also analyzed the involvement of a caspase cascade in MC-LR induced apoptosis. After 24 h of exposure to 8, 16, and 32 μg/mL of MC-LR, the activation of caspase-3 and cleaved-caspase-3 was detected at a concentration as low as 8 μg/mL. As caspase-3 can be activated by one of the initiator caspases, caspase-9, we further attempted to assess the activation of caspase-9, as determined by the induction of cleaved-caspase-9 as well as caspase-9. Both caspases were activated after treatment with 8 μg/mL of MC-LR (Figure 3). These findings indicated that MC-LR activated both initiator and executioner caspases in a concentration-dependent manner.

### MC-LR-induced intracellular ROS generation and the rescuing effects of NAC

**Figure 4:**
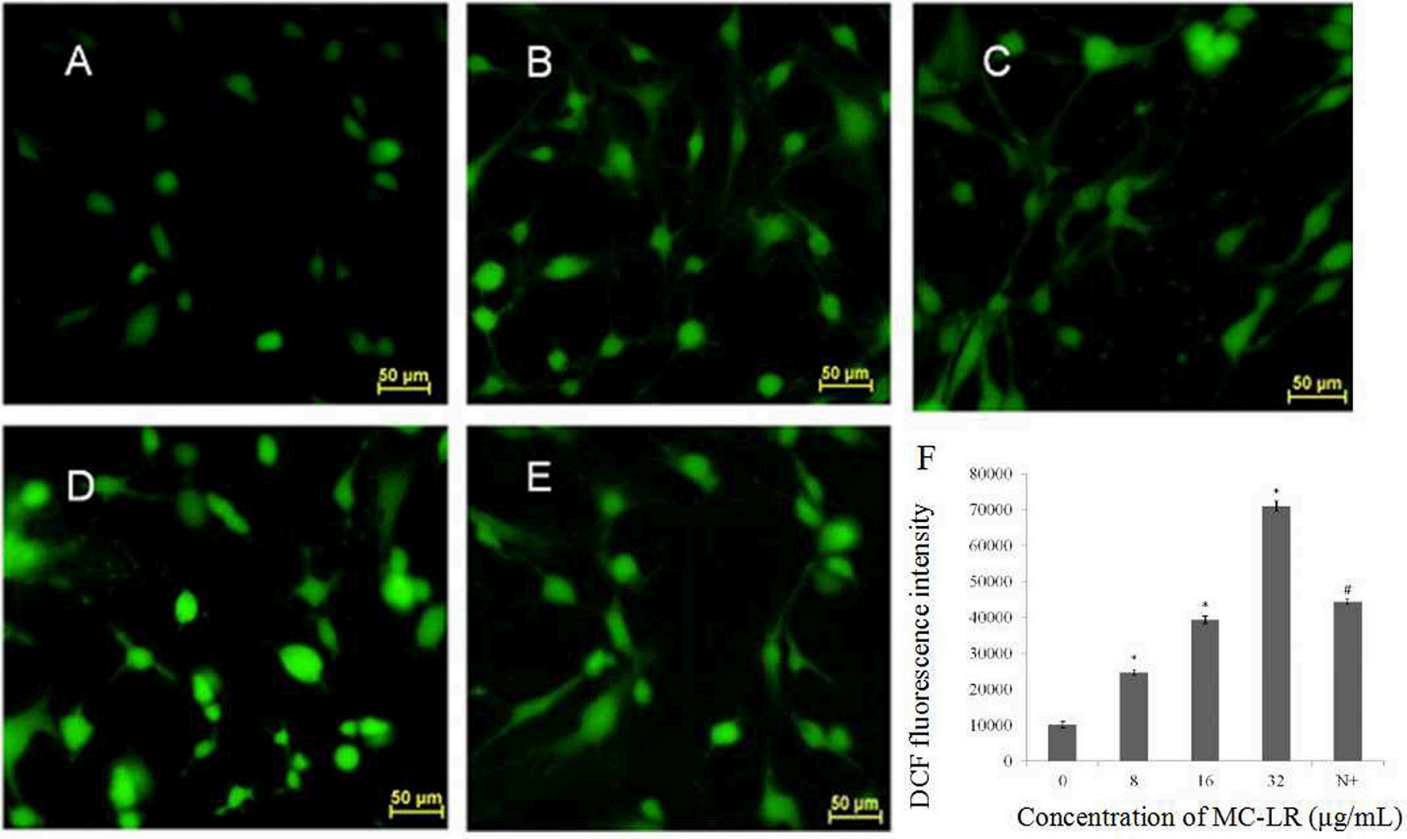
**Effects of MC-LR on ROS content in rat Sertoli cells were observed by fluorescence microscope (200×) and monitored by measuring the DCF fluorescence intensity *via* Flow cytometry. (A)** Control group; **(B)** 8 μg/mL MC-LR; **(C)** 16 μg/mL MC-LR; **(D)** 32 μg/mL MC-LR, **(E)** 10 mM NAC + 32 μg/mL MC-LR; Bar = 50 μm. **(F)** The qualitative representative images. Fluorescence intensity is presented as mean ± SEM of three independent experiments. ^*^P < 0.05 vs. control group; ^#^*P* < 0.05 vs. 32 μg/mL MC-LR.

**Figure 5:**
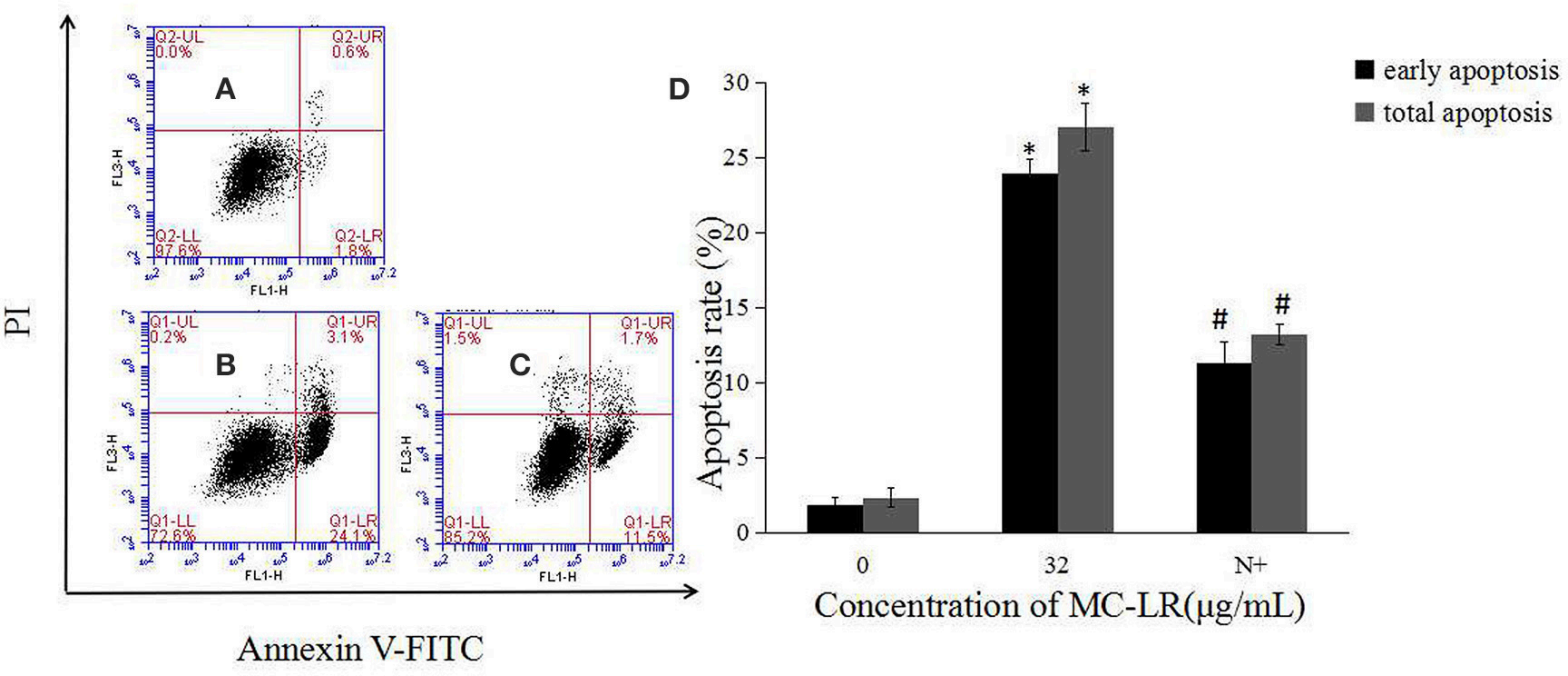
**Effects of NAC pretreatment on apoptosis rate of rat Sertoli cells. (A–C)** Cell apoptosis rate was determined by flow cytometry. **(A)** Control group; **(B)** 32 μg/mL MC-LR; **(C)** 10 mM NAC+32 μg/mL MC-LR; **(D)** Data were expressed as mean ± SEM. N+, 10 mM NAC + 32 μg/mL MC-LR. ^*^P < 0.05 vs. control group; ^#^*P* < 0.05 vs. 32 μg/mL MC-LR.

Mitochondria, which are both a major source of intracellular ROS and a primary target for ROS, play a key role in the regulation of apoptosis. To determine the involvement of ROS during MC-LR-induced apoptosis, Sertoli cells were exposed to 8~32 μg/mL of MC-LR for 24 h, and the intensity of DCF fluorescence was observed via fluorescence microscope. As shown in Figures 4A–D, MC-LR treatment induced intracellular ROS fluorescence intensity dose-dependently. However, when cells were treated with a mixture of MC-LR and NAC (with 1 h of preincubation), ROS intensity was decreased as compared to cells that were treated with MC-LR alone (Figure 4E). Moreover, ROS expression levels were detected by flow cytometer, and the results showed that ROS level was increased dose-dependently after MC-LR treatment, but decreased when cells were treated with a mixture of MC-LR and NAC (Figure 4F). Meanwhile, the inhibitory effect of NAC on MC-LR-induced apoptosis was also confirmed. As shown in Figures 5A–C, MC-LR treatment induced apoptosis obviously, however, when cells were treated with a mixture of MC-LR and NAC (with 1 h of preincubation), apoptosis ratio was decreased as compared to cells that were treated with MC-LR alone. The percent of early apoptosis cells was remarkably reduced (Figure 5D).

### The anti-apoptotic effect of NAC is mediated via mitochondrial caspases

**Figure 6:**
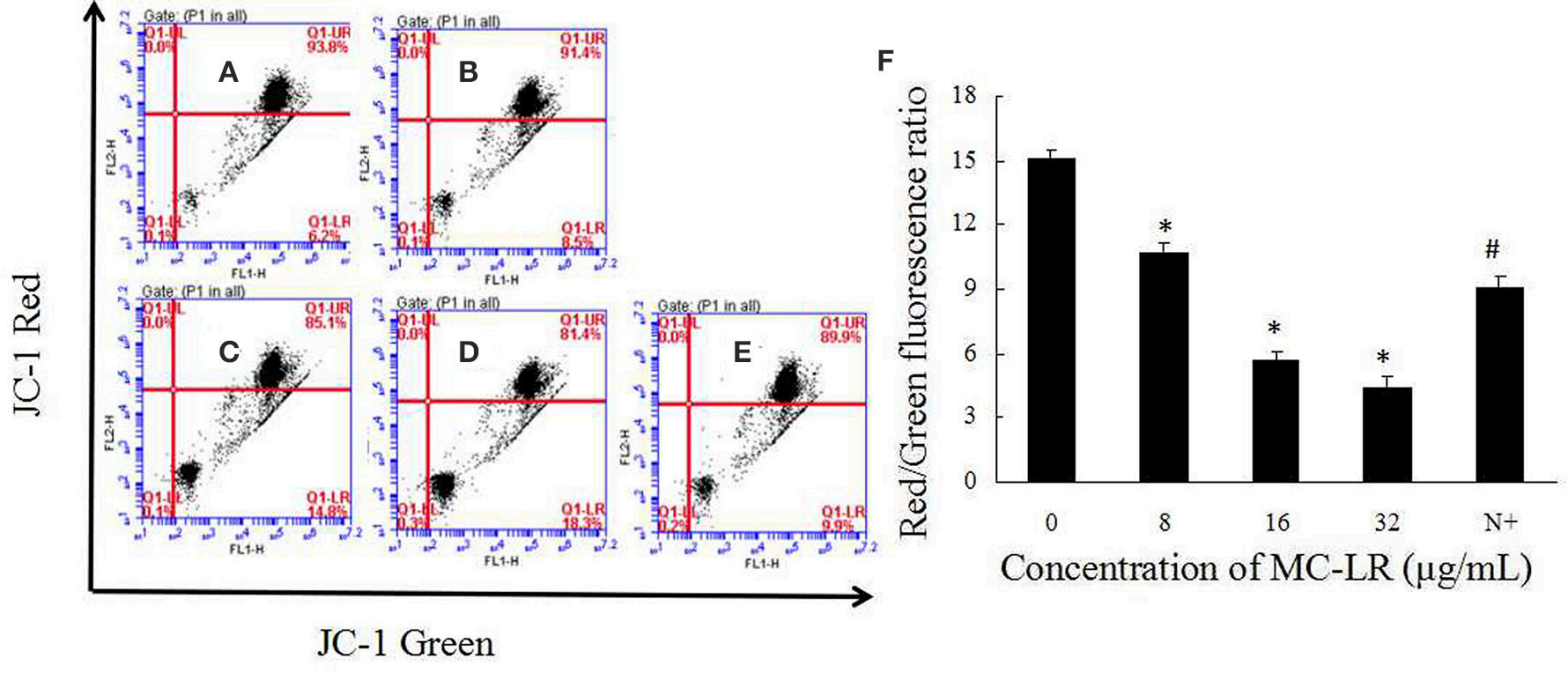
**Effects of MC-LR on MMP in rat Sertoli cells were detected using JC-1 probe via FACS analysis. (A–E)** MMP was determined by flow cytometry. **(A)** Control group; **(B)** 8 μg/mL MC-LR; **(C)** 16 μg/mL MC-LR; **(D)** 32 μg/mL MC-LR; **(E)** 10 mM NAC+32 μg/mL MC-LR; **(F)** Data are presented as mean ± SEM. N+, 10 mM NAC+32 μg/mL MC-LR. ^*^P < 0.05 vs. control group; ^#^*P* < 0.05 vs. 32 μg/mL MC-LR.

It has been proposed that ROS can mediate intracellular signaling cascades, which could induce MMP collapse and mitochondrial dysfunction. We attempted to elucidate the effect of MC-LR on MMP via FACS analysis using the mitochondrial-specific probe JC-1. Sertoli cells were exposed to 8~32 μg/mL of MC-LR for 24 h, and the MMP was observed via fluorescence microscope. As shown in Figures 6A–E, loss of MMP was also observed in the MC-LR-exposed cells. Furthermore, the relationship between ROS production and changes in MMP was characterized with the ROS scavenger NAC. As expected, the decrease in MMP was blocked by co-treatment with NAC (Figure 6F).

**Figure 7:**
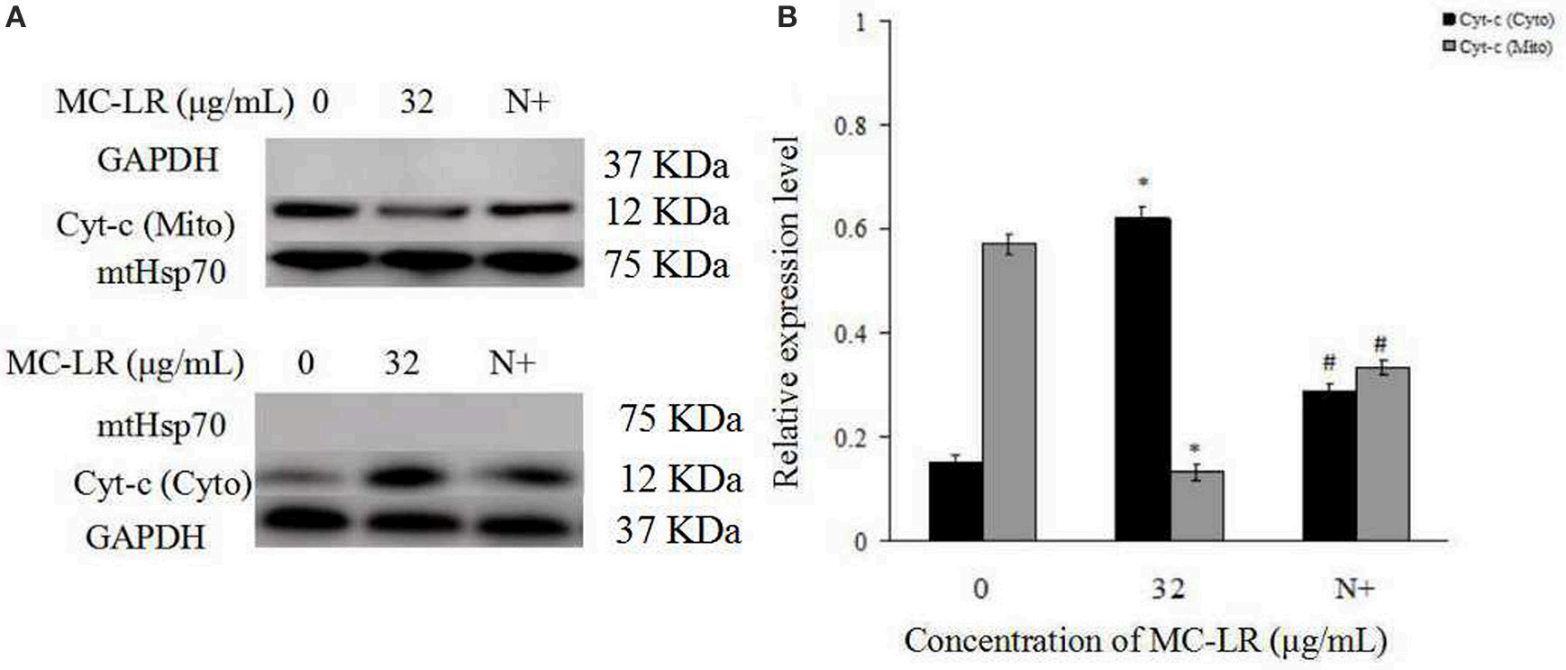
**Effects of NAC pretreatment on distribution and protein expression of cytochrome c (Cyt c). (A)** Rat Sertoli cells were treated with 32 μg/mL MC-LR or NAC + 32 μg/mL MC-LR. The expression of Cyt c and GAPDH in the mitochondrial fraction was tested by western blotting analysis; Cyt c and Hsp70 protein in the cytoplasmic fraction was tested by western blotting analysis. Cyto, cytosol; Mito, mitochondria; N+, 10 mM NAC+32 μg/ml MC-LR. GAPDH was used as the cytosolic control and mitochondria Hsp70 was used as the mitochondrial control. **(B)** Data were presented as mean ± SEM of three independent experiments. ^*^P < 0.05 vs. control group; ^#^*P* < 0.05 vs. 32 μg/mL MC-LR.

**Figure 8:**
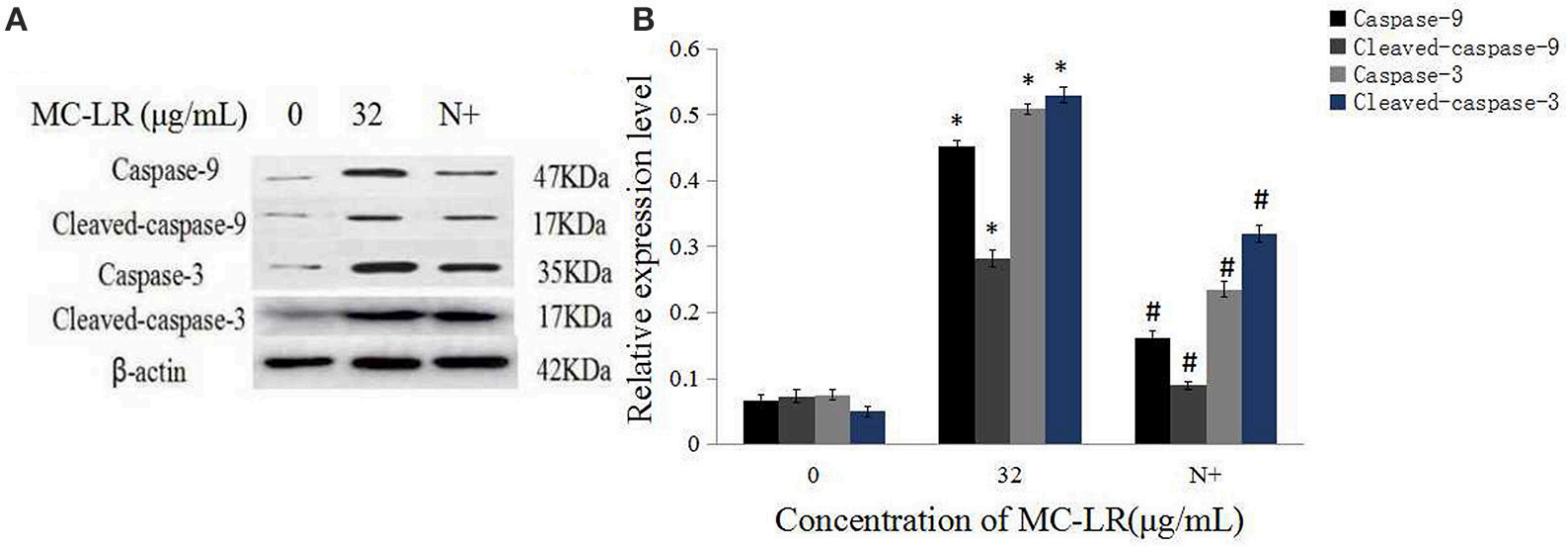
**Effects of NAC pretreatment on expression of caspase proteins. (A)** Rat Sertoli cells were treated with 32 μg/mL MC-LR or NAC+32 μg/mL MC-LR and expressions of caspase-9, cleaved-caspase-9, caspase-3, and cleaved-caspase-3 proteins were tested by western blotting. N+, 10 mM NAC+32 μg/mL MC-LR. β -actin was used as the internal control. **(B)** Data are presented as mean ± SEM of three independent experiments. ^*^P < 0.05 vs. control group; ^#^*P* < 0.05 vs. 32 μg/mL MC-LR.

Mitochondria Cyt c, an important intermediate in intrinsic mitochondrial apoptosis, plays a well-documented role in activating caspase-9 and caspase-3 to execute cell death. We first separated the mitochondria from the cytoplasm of the cells. Then, the expressions of GAPDH in the mitochondrial fraction, and Hsp70 in the cytoplasm were detected. The results showed that there was no GAPDH band in the mitochondrial fraction nor as there Hsp70 band in the cytoplasm (Figure 7). This demonstrated that the subcellular fractionation was successful. As shown in Figure 7, in accordance with inhibition of ROS generation following co-treatment with MC-LR and NAC, Cyt c release in the mitochondria of Sertoli cells was down-regulated significantly (*P* < 0.05) compared with the non-NAC group. In addition, the activation of capase-3, caspase-9, cleaved-caspase-3, and −9 were reversed in the presence of NAC (Figure 8). These observations indicate that MC-LR-induced apoptosis can be inhibited by NAC through mitochondrial cascades.

## Discussion

The decline in fertility of animals and humans over the past few decades that is potentially linked to environmental exposure has generated public health interest (Toppari et al., 1996; Jurewicz et al., 2009; Chen et al., 2011). Sperm quality and testicular function in male mice decrease during chronic low-dose exposure to MC-LR. Many of the experimental data suggested that the reproductive system is the second most important target organ for MCs besides the hepatopancreas/liver (Chen and Xie, 2005; Zhang et al., 2007, 2009; Papadimitriou et al., 2012). In China, both field and laboratory data have raised questions and great concerns about the potential reproductive toxicity of MCs, especially on the male reproductive system. Several studies have verified that MCs accumulate in Sertoli cells and spermatogonic cells but not leydig cells *in vivo* or *in vitro* (Wang et al., 2012; Xiong et al., 2014).

Previous reports have demonstrated that MC-LR-associated reproductive cytotoxicity is intimately related to apoptosis induction (Zhang et al., 2011; Wang X. T. et al., 2013). In our study, we attempted to characterize the molecular events occurring at the earliest stages of apoptosis induction by MC-LR. It is well-accepted that the intrinsic caspase signal activation is mediated *via* mitochondrial dependent pathway. Here, the present data demonstrated that ROS generation is the primary effect caused by MC-LR-induced apoptosis in rat sertoli cells, with subsequent MMP collapse, Cyt c release, eventually leading to the involvement of other mitochondria-mediated pathways at indicated dosages. This is in accordance with other studies using a similar culture model, where MC-LR induced a dose-dependent alteration in apoptosis-associated protein (Li and Han, 2012; Chen et al., 2013). We also determined that the effects of NAC treatment on MC-LR-induced apoptosis occurred via the suppression of ROS generation.

The doses of MC-LR used in this study were selected based on our previous researches and evaluated independently through statistical analysis (Zhang et al., 2011). MC-LR at 8–32 μg/mL effectively inhibited cell proliferation and induced apoptosis in rat Sertoli cells. It is well-known that the primary function of testicular Sertoli cells is to support and nourish sperm cells, thus playing an important role in spermatogenesis (Griswold, 1998; Kaur and Bansal, 2004). Our results showed that MC-LR exposure resulted in the loss of support by testicular Sertoli cells to the spermatogenesis process, which may contribute to sperm cell apoptosis.

Among the apoptotic pathways, mitochondria are recognized as the central executioners. The intrinsic apoptotic pathway is mediated via mitochondria membrane permeabilisation and release of Cyt c into the cytoplasm. Cytochrome c then forms the apoptosome, which subsequently stimulates the caspase cascade through caspase-9 (Kroemer et al., 2007). Recently many researches have shown that MC-LR could induce various cell lines apoptosis through different apoptotic signaling pathways, ultimately leading to the activation of caspases (Chen et al., 2016). In agreement with previous reports, our current study also showed that a pan-caspase inhibitor zVADfmk inhibited MC-LR-induced Sertoli cell death, and MC-LR treatment activated caspase-3 and −9, cleaved-caspase-3, and −9. In addition, several apoptosis-related genes such as Bcl-2, Bax and Caspase-3 are involved in the regulation of testicular cells apoptosis induced by MC-LR in mice testis (Wang X. T. et al., 2013). Taken together, these data demonstrated that MC-LR-induced reproductive toxicity is mediated via activation of intrinsic caspase signal *in vivo* and *in vitro* (Li et al., 2008).

NAC is known to regulate ROS generation, thus modulating the early stages of apoptosis. ROS accumulation has also been demonstrated to induce the depolarization of the mitochondrial membrane. This eventually results in an increase in the level of ROS along with the content of other proapoptotic molecules in the cytosol (Song et al., 2008; Chuang et al., 2011). We found that ROS generation and MMP collapse induced by MC-LR treatment were regulated in a concentration-dependent manner, which suggested that MC-LR is capable of inducing sustained mitochondrial dysfunction. Moreover, pretreatment with ROS scavenger NAC was able to inhibit the release of Cyt c, subsequently reducing the expression of caspase-3 and −9, which indicated that blockade of this ROS-mediated signal cascades may partly attenuate the toxic effects of MC-LR. It is generally accepted that apoptosis can be induced by catalyzing ROS to execute oxidative modification of cellular components, interfering with intracellular redox balance, and/or modulating redox-related signal pathways (Liu et al., 2010; Panieri and Santoro, 2015). In another study from the Zhang group, NAC effectively blocked MC-LR-induced apoptosis of CHO cells through inhibition of ROS generation and partly through restoration of enzymatic antioxidant activity (Xue et al., 2015). It is noteworthy that our frontier study explicitly demonstrated the protective effects of NAC on reproductive toxicity by MC-LR, indicating that NAC can attenuate cell injury and apoptosis. The study also showed that ROS generation contributed to the mitochondria-mediated caspase-dependent apoptosis induced by MC-LR in Sertoli cells.

In conclusion, the present study demonstrated that ROS is essential for MC-LR-induced caspase–mediated apoptosis in Sertoli cells. MC-LR could induce sustained ROS generation in Sertoli cells, and subsequently trigger mitochondrial caspase-dependent death pathway. Conversely, NAC treatment partly inhibited the MC-LR-induced apoptosis via the down-regulation of ROS-mediated signal cascades.

## Author contributions

HH Study Design, data interpretation, manuscript preparation, literature search. CL Data collection, data interpretation, literature search, manuscript preparation. XF Statistical analysis, data Interpretation, manuscript preparation. SZ Data collection, literature search. YX Data collection, literature search. YL Data collection, literature search. LX Data collection. XC Data collection, literature search. HZ Study design, data interpretation, manuscript preparation, funds collection.

### Conflict of interest statement

The authors declare that the research was conducted in the absence of any commercial or financial relationships that could be construed as a potential conflict of interest.
