# Supplementary material for: Microcystin-LR Induced Apoptosis in Rat Sertoli Cells via the Mitochondrial Caspase-Dependent Pathway: Role of Reactive Oxygen Species
Source: Front Physiol. 2016 Sep 9;7:397. doi: 10.3389/fphys.2016.00397 (PMC5016609; doi:10.3389/fphys.2016.00397)
Supplement: Supplementary file 1 [file DataSheet1.PDF]

Supplemental information

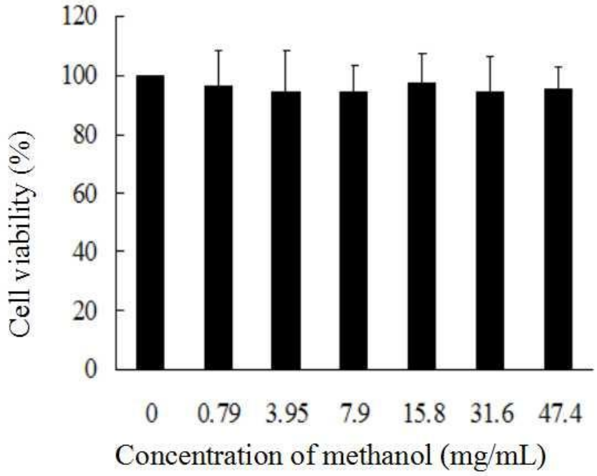

Figure S1 Effects of methanol on the cell viability. Cells were exposed to methanol at different concentrations (0, 0.79, 3.95, 7.9, 15.8, 31.6 and 47.4 mg/ml) for 24 h. The optical density (OD) was detected with CCK-8 assay. Data were presented as mean ± SEM of triplicate independent experiments.
